# Immunohistochemical localization of mdm-2, p27^Kip1 ^and bcl-2 in Warthin's tumor of the parotid gland

**DOI:** 10.1186/1746-1596-4-14

**Published:** 2009-05-16

**Authors:** Ehab S Abd-Elhamid, Marwa M Elshafei

**Affiliations:** 1 Oral Pathology Department, Faculty of Dentistry, Ain Shams University, Cairo, Egypt; 2 Oral Pathology Department, Faculty of Dentistry, Misr International University, Cairo, Egypt

## Abstract

**Background:**

Warthin's tumor is a benign monomorphic adenoma with unclear origin that almost occur exclusively in the parotid gland. Etiology of Warthin's tumor as well as its malignant potential are still unclear. Therefore immunohistochemical assessment of Warthin's tumor may be useful to detect its origin or its malignant transformation potential.

**Aims and objectives:**

The present study aims to investigate the immunohistochemical expression of murine double minute-2 (mdm-2), p27^Kip1 ^and B cell lymphoma-2 (bcl-2) in Warthin's tumor of parotid gland and also to clarify the role of these proteins in the behavior of that tumor.

**Methods:**

Twenty paraffin blocks of cases previously diagnosed as Warthin's tumor were collected for immunohistochemical staining with primary antibodies against mdm-2, p27^Kip1 ^and bcl-2 using streptavidin-biotin immunoperoxidase staining system.

**Results:**

All cases showed immunopositivity for mdm-2 and p27^Kip1 ^while 18/20 showed bcl-2 immunopositivity. Both layers of the neoplastic epithelial cells that line the cystic spaces showed immunopositivity with all antibodies used. Goblet cells were mdm-2 immunonegative while myoepithelial cells were p27^Kip-1 ^immunonegative. Areas of epithelial proliferation that formed buds were p27^Kip-1 ^and bcl-2 immunopositive.

**Conclusion:**

Mdm-2 played a tumor-suppressor role that might be implicated with the benign behavior of Warthin's tumor. The mutual expression of both p27^Kip1 ^and bcl-2 suggested a protective role of these slowly proliferating cells from apoptosis to maintain their survival and elevated bcl-2 expression offers a significant protection against p27^Kip1^-mediated apoptosis.

## Background

Warthin's tumor is classified as a benign monomorphic adenoma of salivary gland that occurs exclusively in the parotid gland. Its etiology is still controversial; some authors suggested a bi-layered excretory ductal structure of the neoplastic epithelium in Warthin's tumor, as a result of hyperplastic process of the glandular epithelium that interacts with the excessive lymphoid tissue of the stroma [1]. Some authors consider it as a tumor like condition, not a monomorphic adenoma as it resulted from the proliferation of salivary gland ductal cells that were entrapped in parotid lymph nodes during embryonal life [2]. Although, other investigators detected, rare, histological change from Warthin's tumor to mucoepidermoid carcinoma that may be implicated in squamous or goblet cell metaplasia of epithelial cells. It is considered that the neoplastic cells of Warthin's tumor acquire malignant genotypes simultaneously with this dual differentiation leading to the formation of mucoepidermoid carcinoma, mostly low grade [3,4]. Therefore immunohistochemical assessment of Warthin's tumor may be useful to detect its origin or its malignant transformation potential.

Mdm2 is identified as an evolutionary conserved gene and as a dominant transforming oncogene on the long arm of the chromosome twelve. It encodes a number of alternatively spliced mRNAs that give rise to proteins ranging in size from 40 kilo Dalton (KDa) to 90 KDa: p90, p57–58, p74, p76 and p85 [5].

Mdm2 performs several functions; some of them are related to p53. By binding to p53, mdm2 has at least two functions: first it can target p53 for ubiquitin-degradation. The process of ubiquitination is subjected to a feedback loop as mdm2 protein binds to p53 to be degraded. This lowers the concentration of p53 and reduces transcription of mdm2 gene, closing the feedback loop and allowing p53 levels to rise again [6].

Second, mdm2 can also bind to the acidic activation domain of p53, concealing it from the transcriptional machinery i.e. blocking the ability of p53 to be recruited into active transcriptional complexes [7].

P90, the major product of mdm2, is now considered to form a tight complex with both the wild-type and mutant p53 tumor-suppressor gene product and inactivate wild-type p53 function by masking the N-terminal acidic transactivating domain of p53 protein, indicating that abnormalities of mdm2 gene may be closely associated with tumorigenesis and/or tumor development [8].

The smaller mdm2 protein, p76, increases the level of p53 by blocking the function of p90. Therefore, although, p90 and p57 bind to p53 and inhibit its nuclear accumulation, p74/76 can stabilize p53 by inhibiting the ability of p90 to stimulate its degradation without affecting the p90/p53 interaction [8].

Mdm2 may have a role in preventing apoptosis, either through inactivation of p53 or through some other mechanism independent of p53 [6]. On the other hand, it may down-regulate the anti-apoptotic protein Bcl-2 [9].

Inactivation of mdm2-induced G0/G1 arrest may contribute to tumor development [10], hence, mdm2 appears to play dual roles, as a tumor suppressor and as an oncogene, depending on the levels of mdm2 being expressed in the cell [11].

Mantesso et al [12] and Schlott et al [13] reported that mdm2 gene seems to be an early event related to the development of benign salivary gland tumors and play a part in tumor progression in salivary gland neoplasms.

P27^Kip1^, a member of kinase inhibitory protein (KIP) family was identified as an inhibitory protein induced by tansforming growth factor-β (TGF-β) that played an important role in differentiation, senescence and cellular responses to negative growth-regulatory cytokines and to DNA damage and hence plays an essential role in the proliferative arrest that accompanies differentiation as well as is integrally involved in ensuring an orderly progression through the cell cycle [14].

Levels of p27^Kip1 ^are high in normal quiescent cells (G0/G1 cells) but decrease in cycling cells, rapidly after mitogenic stimulation, when it gets sequestered into excess cyclin-cyclin-dependent kinase complexes. It remains low thereafter in actively proliferating cells [15].

Different mechanisms appear to regulate apoptosis in resting or cycling cells; deregulated activation of cyclin-dependent kinases results in apoptosis in resting cells, whereas cyclin-dependent kinase inhibition induces apoptosis in cycling cells. Therefore, apoptosis may result from activation of pathways that promote apoptosis upon inappropriate expression of cell cycle regulatory molecules. P27^Kip1 ^functions at a critical switch point where growth arrest is followed either by differentiation or apoptosis [16].

Apoptosis is modulated by several proteins or antigens such as the bcl-2 family and p53. Regulation of apoptosis is dependent on interactions between effector and suppressor molecules. Deregulation of the genes controlling apoptosis may contribute to the process of tumourigenesis by reducing the rate of cell death and allowing the accumulation of other genetic defects [17].

Bcl-2 emerged as a new type of proto-oncogene that suppresses cell death rather than stimulating proliferation. However, it does not inhibit apoptosis occurring in all circumstances as it failed to block apoptosis induced by cytotoxic T-lymphocytes [18].

The involvement of bcl-2 in p53-induced apoptosis was first suggested by the observation that p53-induced cell death can be prevented by bcl-2 expression. Wild-type p53-dependent apoptosis occurs at least partly through its suppressive effect on bcl-2 expression or its function. Support for this has come from in vitro studies of lymphoid cells where ectopic p53 was shown to down-regulate endogenous bcl-2 expression while simultaneously increasing the expression of bax [19].

The aim of the present study is to investigate the immunohistochemical expression of mdm-2, p27^Kip1 ^and bcl-2 in Warthin's tumor of parotid gland and also to clarify the role of these proteins in the behavior of that tumor.

## Method

### Tissue samples

Twenty paraffin blocks of cases previously diagnosed as Warthin's tumor were collected from the archives of the Oral Pathology Department, Faculty of Dentistry, Ain Shams University. Haematoxylin and Eosin stained sections were prepared for each specimen to confirm the diagnosis. From each of the selected paraffin blocks three sections of 4 μm thick were cut and mounted on positively charged slides (Biogenex Corp, California, USA) for immunohistochemical staining.

### Reagents

Monoclonal antibodies against mdm2, p27^Kip1 ^and bcl-2 were used. These antibodies recognized their corresponding proteins in paraffin-embedded sections. Monoclonal IgG-Kappa antibody against mdm2 was purchased from (Zymed Laboratories Inc., USA). It recognizes epitope located within the amino acid 26–169 of the human mdm2 protein. While monoclonal antibody against p27^Kip1 ^was purchased from (DAKO Corporation, USA) and monoclonal antibody against bcl-2 was purchased from (Zymed Laboratories Inc., USA).

A streptavidin-biotin immunoperoxidase staining system was used for immuno-detection. It included the following reagents:

• Monoclonal Linking Reagent: biotinylated anti-mouse immunoglobulin

• Streptavidin Enzyme Label: horseradish peroxidase-conjugated streptavidin

• Buffered Substrate: Hydrochloric acid (HCl) buffer, pH 7.5

• Enzyme Substrate: 0.6% hydrogen peroxide

• DAB Chromogen Concentrate: 3.3' diaminobenzidine in chromogen solution

• Buffered Wash Solution: phosphate buffered saline (PBS), 0.1 mol/l, pH 7.4 ± 2

The detection systems were purchased from DAKO Corporation, USA and Zymed Laboratories Inc., USA.

The retrieval solution was purchased from BioGenex, USA.

### Immunohistochemistry

Sections were deparaffinized in hot xylene. Rehydration of slides was performed by immersing slides in descending alcohol concentrations and then washed in distilled water. The sections were rinsed in two washes of phosphate-buffered saline for 5 minutes each (PBS; pH 7.4). For staining with mdm2 antibody, the sections were microwave antigen retrieved with citrate buffer solution (pH 4.8 for 15 minutes). P27 ^Kip-1 ^and bcl-2 antibodies required antigen retrieval by microwave processing in 0.2 M glycine buffer (Glyca, pH 6.2 ± 0.2, for 15 min.). Sections were again rinsed in two washes of PBS. Endogenous peroxidase activity was blocked with 3% hydrogen peroxide in water. The primary antibodies anti-mdm2, anti-p27 ^Kip-1^, and anti-bcl-2 were applied and the slides were incubated in a humid chamber overnight at room temperature. The sections were then washed in PBS. The secondary (Link) antibody (Biotinylated anti-mouse IgG, dilution 1:25) was then applied and the slides were incubated for 20 minutes at room temperature. After washing with PBS, the slides were incubated with peroxidase-labelled streptavidin complex for 20 minutes at room temperature, and then washed with PBS. The slides were then incubated with a solution of 3% diamino-benzidine (DAB) as the chromogen for 20 minutes. Finally, the tissues were lightly counter stained with Mayer's Haematoxylin and mounted with DPX permanent mounting medium.

### Assessment

In this study, the immunostaining of each antibody was assessed in 5 serial sections from each specimen. For mdm2, tumor cells were considered immunopositive when they displayed a brownish nuclear and/or cytoplasmic immunoreactivity, p27 ^Kip-1 ^immunostaining was considered positive when tumor cells showed a nuclear immunoreactivity while bcl-2 was considered positive if the tumor cells showed cytoplasmic or perinuclear localization of immunoreactivity.

## Results and discussion

### Mdm2 Immunohistolochemical Results

**Figure 1:**
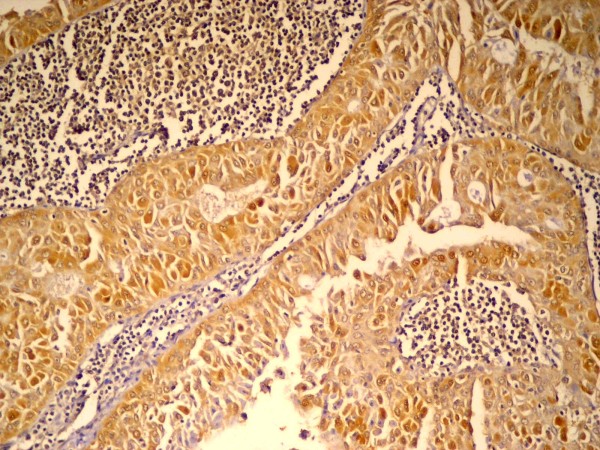
**immunopositive epithelial cells forming papillary growth**. The C.T. stroma is negative (anti-mdm2 ×100).

**Figure 2:**
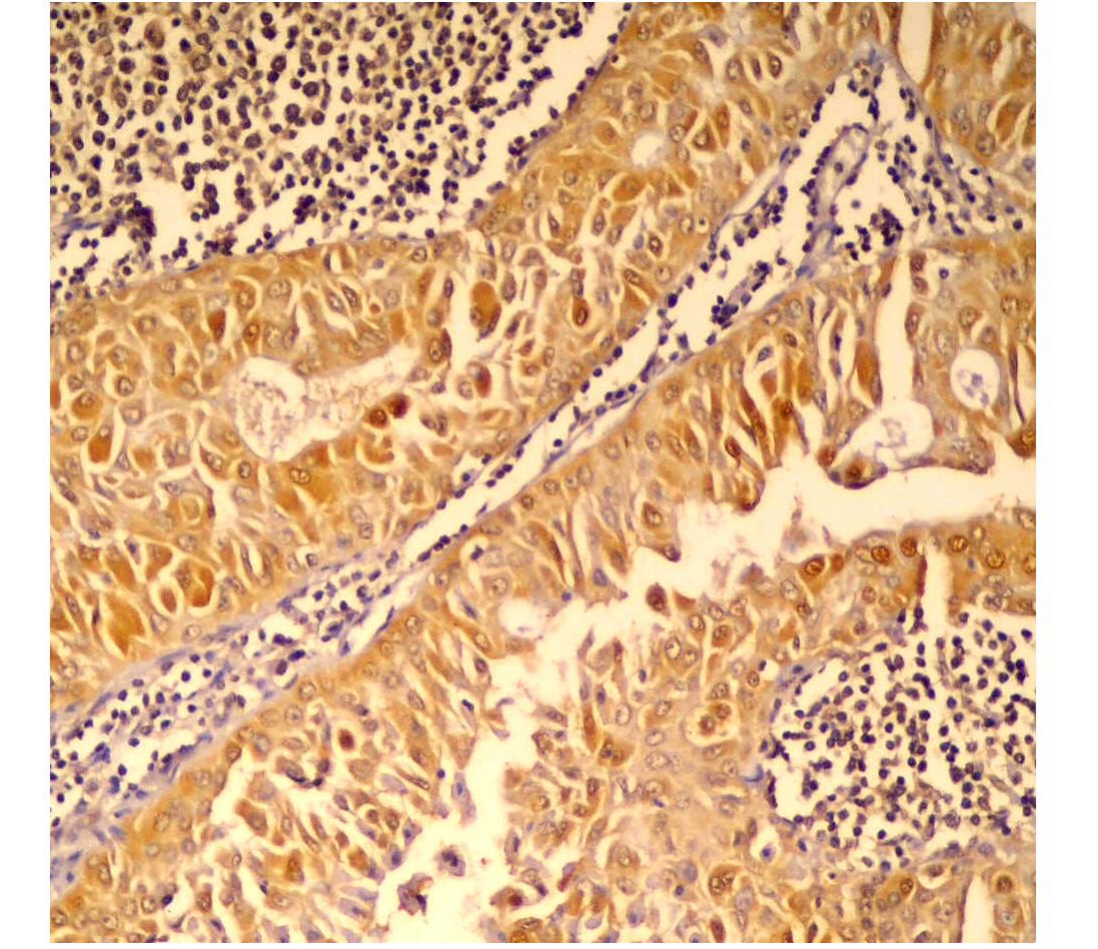
**Higher magnification of figure 1 showing cytoplasmic immunopositivity in both basal and luminal cells**. Goblet cells are immunonegative (anti-mdm2 ×200).

All specimens were positively stained with mdm2. Most of the neoplastic epithelial cells forming papillae showed cytoplasmic immunopositivity, both the cuboidal basal cells and the columnar luminal cells (fig. 1), while goblet cells were immunonegative (fig. 2).

### P27^Kip-1 ^Immunohistolochemical Results

**Figure 3:**
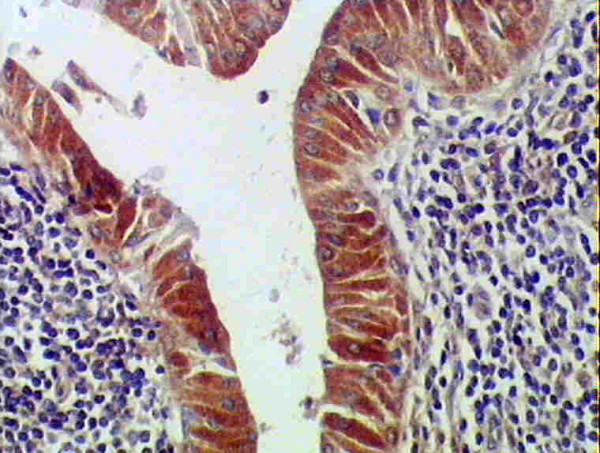
**immunopositive neoplastic epithelial cells forming papillary growth**. The C.T. stroma is negative (anti-p27 ×200).

**Figure 4:**
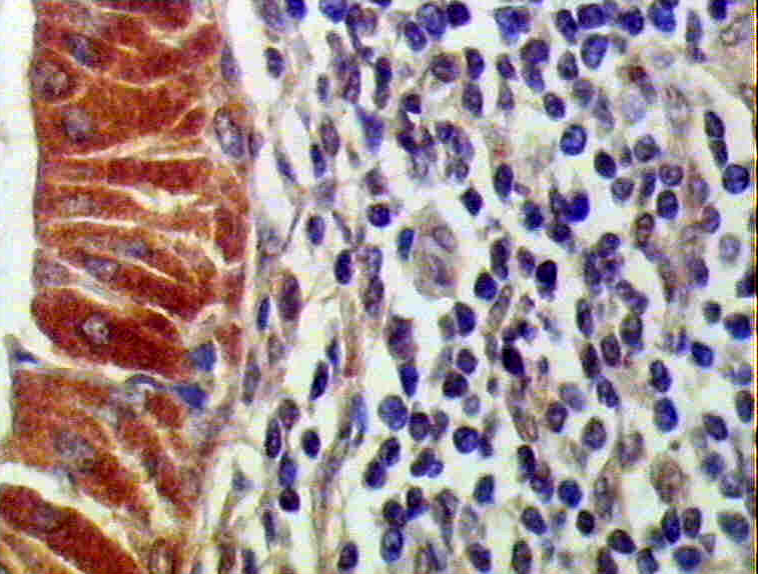
**Higher magnification of figure 3 showing intense immunoreactivity in both layers of neoplastic epithelial cells**. Some cells are showing both nuclear and cytoplasmic immunopositivity (anti-p27 ×400).

All specimens showed positive immunoreactivity. Both layers of the neoplastic epithelium (basal and columnar cells) revealed positive nuclear immunostaining, surrounded by a negatively stained stroma (fig. 3). Basal epithelial cells showed a faint immunppositive reaction. However, a combination of cytoplasmic and nuclear staining was observed (fig. 4). Myoepithelial cells were negatively stained (fig. 4).

### Bcl-2 Immunohistolochemical Results

**Figure 5:**
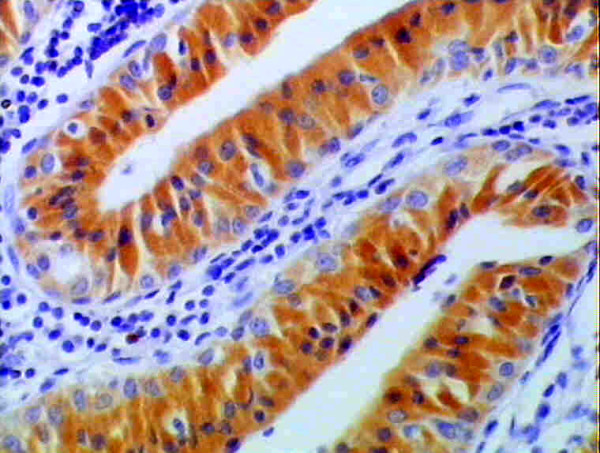
**Positive cytoplasmic immunostaining of luminal cells and few basal epithelial cells**. Myoepithelial cells are immunonegative. The surrounding stroma is also negative (anti-bcl2 ×200).

**Figure 6:**
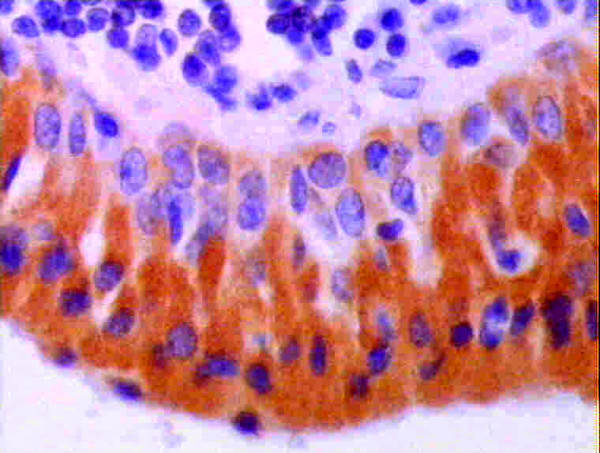
**Higher magnification of figure 5 showing intense positive cytoplasmic immunostaining of luminal cells while few basal epithelial cells are faintly positive**. (anti-bcl2 ×400).

Eighteen out of 20 cases (90%) were positively stained. Both layers of the neoplastic epithelium (basal and luminal cells) showed positive immunoreactivity, with cytoplasmic localization, surrounded by a negatively stained stroma (fig. 5). Few basal cells were faintly positive while others were immunonegative. Myoepithelial and goblet cells were also negative (fig. 6). Areas of budding in epithelial cells showed immunopositive reaction (fig. 6).

## Discussion

This study was aimed to investigate the immunoexpression of mdm2 oncoprotein, p27^Kip1 ^tumor suppressor protein as well as bcl-2 anti-apoptotic protein in Warthin's tumor as a trial to clarify the possible role of these families in the pathogenesis of this unique tumor of salivary glands.

Earlier studies focused on the analysis of p53 gene in tumors of the salivary glands; however the connection between salivary gland carcinogenesis and the mdm2 oncoprotein was still elusive [8]. Some authors demonstrated that not only p53 gene is a potential target in neoplastic transformation, but also genes involved in the regulation of its function since mdm2 can potently regulate p53, and functions as an oncogene in the process of cell transformation [20]. Some authors considered both nuclear and cytoplasmic staining or exclusively cytoplasmic staining as positive results [21], and this was chosen for assessment of immunopositive reaction of mdm2 in this study, because a strong evidence is now available that mdm2 is an RNA binding protein that can shuttle between the nucleus and the cytoplasm [22], and both mdm2 and p53 proteins contain a nuclear-import and nuclear-export signals (NES) that enable them to be directed into the nucleus and out again towards the cytoplasm [7]. This NES of mdm2 is essential for p53 degradation by interacting with cytoplasmic proteasomes, where p53 is specifically degraded [23].

Overexpression of mdm2 protein in glandular epithelium leads to abnormal development, altered histological structure and eventually to overt epithelial neoplasia [21]. Mdm2 is considered an oncoprotein through its p53-dependent and p53-independent functions, therefore the expression of mdm2 in both epithelial cell layers in Warthin's tumor might indicate a high proliferation rate of this tumor.

But since Warthin's tumor is reported to be a benign, non-aggressive tumor [24], therefore the increase amount of mdm2 in these lesions may be attributed to the expression of a smaller protein of the mdm2 molecule called p74/76 that binds to p53 and doesn't inhibit its action or even preserve its presence rather than degrade it. This unique role of p74/76 is mediated through binding with p90 leading to inhibition of its function.

Brown et al [25] stated that the human oncoprotein mdm2 arrests the cell cycle and elimination of its cell cycle inhibitory function induces tumorigenesis. They attributed this finding to the dual role of mdm2, which suggests that full-length mdm2 can induce growth arrest in some cell lines, including some normal cells. Only elimination or inactivation of the mdm2-induced G0/G1 arrest may contribute to one of the steps of tumorigenesis. Some tumor-derived cells are partially insensitive to the mdm2-mediated cell cycle inhibition and mdm2 can be stably overexpressed in these tumor cells. This dual function of mdm2, suggested by other investigators [26], might explain the high level of expression of mdm2 in Warthin's tumor.

The dual role of mdm2 could depend on the level of mdm2 expressed in the individual cell and the balance of some positive and negative regulators of the cell cycle which is critical for control of cell proliferation and small differences in the levels of any of these proteins may mean large differences in cell activities [26].

Immunohistochemical results of the present study also revealed that Warthin's tumour expressed a high level of p27^Kip1 ^and bcl-2, with p27^Kip1 ^immunopositivity detected in all 20 cases, while 18/20 cases were bcl-2 immunopositive. P27^Kip1 ^protein was detected in both layers of the neoplastic epithelium (basal and luminal) indicating the low proliferation rate of these cells. The more faintly stained basal cells indicated their higher rate of proliferation [27].

On the other hand, bcl-2 immunopositivity was also detected in both layers of the neoplastic epithelium. Areas of budding were also immunopositive indicating the attempt of this protein to maintain the survival of these more proliferative cells and that these epithelial cells might play a role as stem cells [28].

The mutual presence of p27^Kip1 ^and bcl-2 proteins in these same cells indicated the protection of these slowly proliferating cells from apoptosis to maintain their survival. This conclusion may put forward that mdm2 in this lesion is not acting as oncogene, but rather act as a tumor suppressor gene or a smaller protein is expressed. High levels of p27^Kip1 ^favor apoptosis, which can be counteracted by other regulatory factors as bcl-2. It was observed that elevated bcl-2 expression did confer significant protection against p27^Kip1^-mediated apoptosis [27]. Bcl-2 protein promotes cell survival in slowly growing tumors and this might decreased the rate of acquiring further defects. Therefore, bcl-2 expression appears to be associated with less aggressive tumor behavior [28].

Bcl-2-Immunonegativity noted in only 2 out of 20 cases of the present study might indicated failure of bcl-2 to block apoptosis in certain circumstances such as apoptosis induced by cytotoxic T-lymphocytes [18], a finding that might indicated a reactive nature of the lymphoid tissue in Warthin's tumor [29].

Some lesions showed proliferating bud at a part of the neoplastic epithelium, that might suggest an aggressive clinical behavior of this lesion or that this lesion may turn into malignancy, however the result of this study attesting the positivity of the neoplastic epithelium for the three antibody used, it is suggested that the proliferating bud is only a part of the non-aggressive lesion as p27^Kip1 ^and mdm2, in this lesion, act as tumor suppressor proteins while the bcl-2 would in part protect these slowly proliferating cells from apoptosis. Therefore, it could be estimated that Warthin's tumor is slowly but continuously proliferating tumor.

## Conclusion

• Mdm-2 could play a tumor-suppressor role that might be implicated with the benign behavior of Warthin's tumor.

• Mutual expression of both p27^Kip1 ^and bcl-2 suggested a protective role of these slowly proliferating cells from apoptosis to maintain their survival but not to increase their malignant potentiality.

• Elevated bcl-2 expression in Warthin's tumor suggests a possible protection against p27^Kip1^-mediated apoptosis.

## Competing interests

The authors declare that they have no competing interests.

## Authors' contributions

EAE participated in the study design, collection of the background references, photomicrography of the immunohistochemical results, displaying the results of the study, writing the discussion of the results, carried out the sequence alignment and drafted the manuscript. MME participated in the study design, collection of the background references, carried out the immunohistochemical technique, participated in displaying the results of the study, writing the discussion of the results and alignment of the references.
